# In Vitro Evaluation of the Effects of Hyaluronic Acid and an Aminoacidic Pool on Human Osteoblasts

**DOI:** 10.3390/biomedicines11030751

**Published:** 2023-03-01

**Authors:** Maria Addolorata Bonifacio, Amalia Cassano, Alessandra Vincenti, Angela Vinella, Fabio Dell’Olio, Gianfranco Favia, Maria Addolorata Mariggiò

**Affiliations:** 1 Department of Precision and Regenerative Medicine and Ionian Area, Medical School, University of Bari Aldo Moro, 70124 Bari, Italy; 2 Department of Interdisciplinary Medicine, University of Bari Aldo Moro, 70124 Bari, Italy

**Keywords:** Normal Human Osteoblast (NHOst) cells, sodium hyaluronate, amino acids, osteoblast differentiation, alkaline phosphatase

## Abstract

The treatment of bone injuries must be timely and effective to improve the chances of full recovery. In this respect, a mix of hyaluronic acid and an amino acidic pool has been marketed to promote soft tissue healing, fastening recovery times. Several studies have reported the in vitro and in vivo influence of hyaluronic acid and amino acids on fibroblasts and keratinocytes, highlighting the enhancement of cell proliferation, motility and cytokines synthesis. Even though the effectiveness of this combination of molecules on bone repair has been described in vivo, to the best of our knowledge, its in vitro effects on osteoblasts still need to be investigated. Therefore, this work describes for the first time osteoblast metabolism, proliferation and in vitro differentiation in the presence of hyaluronic acid and amino acids, aiming at understanding the mechanisms underlying their effectiveness in injured tissue repair. The reported results demonstrate the enhancement of osteoblasts’ metabolic activity and the fastening of cell cycle progression. Furthermore, gene expression studies show a significant increase in differentiation markers, i.e., osteoprotegerin and osteonectin. Finally, alkaline phosphatase activity is also boosted by the combination of hyaluronic acid and aminoacids, confirming the ability of in vitro cultured cells to properly differentiate through the osteogenic lineage.

## 1. Introduction

The aging population is challenged by the spreading of bone diseases, arising from traumatic events, cancers and other age-related pathologies [1,2,3]. Sometimes, full recovery can be achieved through proper surgical treatment, as well as exploiting the most effective medical devices to support tissue repair. Natural polysaccharides are valuable tools to mimic the native extracellular matrix, providing suitable viscoelastic properties to the injured tissue [4,5]. In particular, sodium hyaluronate is one of the main polysaccharides composing the extracellular matrix and is widely exploited as dermal filler, injectable gel for cartilage repair and surgical aid for ophthalmic interventions [6,7,8]. A high molecular weight sodium hyaluronate has been combined with a pool of four amino acids (i.e., proline, lysine, leucine and glycine) in a commercial product able to support the demanding metabolic activity of cells involved in healing processes [9]. Indeed, tissue repair requires the deposition of newly synthesized collagen and proteoglycans to remodel the extracellular matrix; thus, the availability of amino acids within the injured site is particularly helpful [10]. For these reasons, the combination of sodium hyaluronate and amino acids (HA+AA) represents an effective tool to treat wounded tissues, such as oral mucosae [11,12,13]. In addition, the mechanisms underlying the benefits elicited by HA+AA on keratinocytes have been widely explored in previous studies, highlighting that, in the presence of HA+AA, cell migration and synthesis of keratinocyte-derived neuromodulators are significantly improved [14,15]. Moreover, HA+AA is able to induce angiogenic processes, as well as enhance fibroblast proliferation, collagen deposition and biosynthesis of growth factors [16,17]. Concerning bone diseases, the advantages of using HA+AA have been reported through in vivo studies, showing that the consolidation of hand fractures is sped up by HA+AA application immediately after wounding [18]. Furthermore, the clinical efficacy of HA+AA has been proved after tooth extraction, reducing pain and promoting healing [19]. However, the cellular and molecular mechanisms elicited by HA+AA on osteoblast cells still need to be investigated. Hence, in this work, the in vitro effects of HA+AA on normal human osteoblast cells (NHOst) have been explored for the first time. The metabolic activity and the cell cycle of NHOst cells have been analyzed through the MTT assay and flow cytometry techniques, respectively. Then, the gene expression of specific markers of osteoblasts’ maturation has been studied, as well as changes in cell morphology and alkaline phosphatase activity.

## 2. Materials and Methods

### 2.1. Materials

Sodium hyaluronate (HA) and amino acid (AA) powders were kindly gifted by Errekappa Euroterapici S.p.A. (Milan, Italy), which commercializes Aminogam^®^ formulations. NHOst cells (CC-2538), as well as cell culture media (Basal Growth Medium and Osteoblast Differentiation Medium) were purchased from Lonza (Siena, Italy). Fetal bovine serum, trypsin EDTA solution 0.25%, MTT and ALP assay reagents were supplied by Sigma-Aldrich (Milan, Italy), while L-glutamine was provided by Euroclone Ltd. (Milan, Italy). The TRIZol™ reagent, SuperScript™ III First-Strand Synthesis System, Quant-iT™ PicoGreen^®^ dsDNA Assay kit, poly-lysine coated T-25 flasks, 6-well plates and 96-well plates were purchased from ThermoFisher Scientific (Milan, Italy). The ALP staining kit (ab242286) was supplied by Abcam plc (Cambridge, UK).

### 2.2. HA+AA Solution Composition

The HA+AA solution was made of sodium hyaluronate (HA, 1.33% *w*/*v*; MW 1.64 · 10^6^ Da) and a pool of amino acids (AA, 2% *w*/*v*) in sterile distilled water [17]. The pool of AA consisted of 1% *w*/*v* glycine, 0.75% *w*/*v* proline, 0.15% *w*/*v* leucine and 0.1% *w*/*v* lysine HCl. Then, the prepared solution was serially diluted in sterile distilled water.

### 2.3. Cell Culture

Human primary osteoblast cells (NHOst) were cultured as recommended by the supplier (Lonza s.r.l., Rome, Italy) and as described by Rajzer and coworkers [20]. Briefly, cells were seeded in T-25 flasks and cultured at 37 °C and 5% CO_2_ in Osteoblast Growth Medium, supplemented with 10% of fetal bovine serum, 25.5 µg/mL ascorbic acid and a mix of 30 µg/mL gentamicin and 15 ng/mL amphotericin-B. Furthermore, when confluent, NHOst cells were detached with trypsin and split in new flasks. For all of the experiments, cells were exploited at the sixth passage. For NHOst cell gene expression experiments, the basal Osteoblast Growth Medium was supplemented with 200 nM hydrocortisone-21-hemisuccinate and 7.5 mM β-glycerophosphate. NHOst cell synchronization was promoted by adding 2% fetal bovine serum to the Osteoblast Growth Medium.

### 2.4. Metabolic Activity of NHOst Cells

NHOst cells (3 × 10^3^ per well) were seeded in 96-well plates and cultured for 24 h. Then, six dilutions of the HA+AA solution were added in triplicate. After three and four days, the medium with HA+AA was removed and replaced with fresh culture medium containing 10 µL of MTT solution (5 mg/mL), as described elsewhere [21]. The plates were incubated for 3 h at 37 °C; then, the formazan crystals were dissolved adding a solution of HCl 0.01 M and SDS 10% and incubated for 24 h at 37 °C. The Bio-Rad microplate reader model 680 (Bio-Rad Laboratories, Segrate, MI, Italy) was exploited to measure the absorbance at 570 nm. The results are reported as mean ± standard deviation and were obtained via six independent assays.

### 2.5. Cell Cycle Analyses

Flow cytometry experiments were performed using the Epics XL-MCL^®^ Flow Cytometer (Beckman Coulter S.p.A., Milan, Italy) on NHOst DNA to gain insights into the dynamics of cell cycle progression of NHOst cells exposed to Aminogam^®^ solution. Briefly, 3 × 10^5^ NHOst cells were cultured in the presence of HA+AA solution up to three days; then, cell culture medium was replaced with PBS and cells were detached with trypsin 0.025%. The harvested cells were washed with cold PBS and fixed in 70% ethanol for 1 h at 4 °C, as reported in [22]. Then, fixed cells were centrifuged at 850× *g* and stained with a propidium iodide solution of 50 µg/mL, prepared in PBS containing 20 mg/mL of RNase A and 0.05% Triton X-100. Collected data were analyzed using the ModFit LT™ software version 6.0 (Verity Software House, Topsham, ME, USA).

### 2.6. Gene Expression Studies via RT-PCR

For 23 days, NHOst cells (3 × 10^5^) were cultured in T-25 flasks containing basal or supplemented medium and exposed to HA+AA solution. Then, cells were washed thrice with cold PBS and lysed with 2.5 mL of TRIzol™ reagent. Briefly, after 5 min of incubation, cell lysates were harvested and exposed to 0.5 mL of chloroform, and were then centrifuged at 12,000× *g* for 15 min at 4 °C. Then, the upper aqueous phase was transferred in a new tube and mixed with 1.25 mL of isopropyl alcohol. After another centrifugation step, the RNA pellet was resuspended in 75% ethanol. Spectrophotometric measurements were performed to assess the RNA concentration and to choose the proper volume for the reverse transcription of 2 μg of RNA [23]. In this respect, the SuperScript™ III First-Strand Synthesis System was exploited as recommended by the manufacturer. PCR experiments were performed with 1 µL of the synthetized cDNA as a template, following the thermal cycles reported in Table 1. The PCR products were loaded on 4% agarose gels and stained with ethidium bromide. Gel pictures were captured by the Gel Doc 2000 system (Bio-Rad Laboratories, Segrate, MI, Italy) and analyzed using the Quantity One Software v. 4.6.7 (Bio-Rad Laboratories, Segrate, MI, Italy).

### 2.7. Assessment of ALP Activity and Staining

The activity of alkaline phosphatase was assessed after 4, 7, 11, 14, 19 and 23 days of culture on NHOst cell lysates. Briefly, 40 mg of substrate (p-nitrophenylphosphate disodium) was added to 500 µL of alkaline buffer solution and incubated at 37 °C for 1 h. The production of p-nitrophenol was evaluated through absorbance measurements at 410 nm, performed using the Bio-Rad microplate reader model 680 (Bio-Rad Laboratories, Segrate MI, Italy). ALP absorbance values were normalized with the DNA amount evaluated for each tested sample using the PicoGreen^®^ dsDNA assay kit, as reported in [24]. Briefly, NHOst cell lysates were added in 96-well plates containing 100 μL of PicoGreen^®^ reagent, diluted 1:200 in TE buffer. After 5 min of incubation in the dark, fluorescence intensity was measured through a microplate reader (excitation wavelength 485 nm, emission wavelength 525 nm) and compared to a standard curve built with the dsDNA sample provided by the manufacturer. The results were obtained through six independent experiments.

The ALP staining kit (ab242286; Abcam plc, Cambridge, UK) was performed on NHOst cells, untreated or exposed to HA+AA solution, after 19 and 23 days of culture in Differentiation Medium. Cells were cultured in 6-well plates, fixed with a solution of formaldehyde 4% *v*/*v*, washed with Tris buffered saline solution 0.05% *w*/*v* and stained in the dark for 15 min. Images were captured using an Axioplan II microscope (Zeiss, Oberkochen, Germany) and analyzed using Fiji software [25]. The latter was used to calculate the percentage of the substrate area covered by NHOst cells. Briefly, RGB images were converted to grayscale and met the threshold [26]. Then, the area covered by cells was calculated using Fiji’s measurement tool and expressed as a percentage of the overall area. The analysis was repeated on at least three different microscopy fields per each sample type and the results are reported in Figure S2 as mean ± standard deviation.

### 2.8. Statistical Analyses

ANOVA and the post hoc Bonferroni test were exploited to analyze the results of the MTT assay, as well as those of the cell cycle study, ALP activity and staining. Statistical analyses were performed using R software, version 4.0.1. [27].

## 3. Results

### 3.1. Metabolic Activity of NHOst Cells

**Figure 1:**
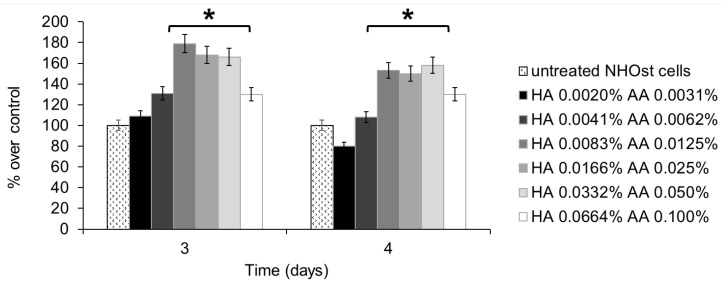
MTT assay performed on NHOst cells after 3 or 4 days of culture, in the presence of different concentrations of HA+AA solutions. Data are expressed as percentage over control, i.e., untreated NHOst cells. Each bar represents the mean of six independent experiments. * *p* < 0.03. HA: sodium hyaluronate, AA: amino acids.

Cell metabolism was assessed using the MTT assay, performed on untreated NHOst cells, as well as on NHOst cells exposed to six different concentrations of HA+AA solutions. Figure 1 shows the results relevant to 3 and 4 days of culture, expressed as percentages of metabolic activity over untreated cells. A slight decrease in cell metabolism, but not statistically significant, was recorded after 4 days of culture in the presence of the lowest concentration of HA and AA. All of the HA+AA solutions have positive effects on NHOst cells’ metabolism, in particular on those solutions where HA amounts range from 0.0083% to 0.0332% *w*/*v* and AA concentrations range between 0.0125% and 0.050% *w*/*v*. These solutions significantly enhance the metabolic activity of NHOst cells after 3 and 4 days of culture (* *p* < 0.03). Between the three most effective solutions, no statistically significant differences were detected. Therefore, the Aminogam^®^ solution with the lowest concentrations of HA and AA (0.0083% and 0.0125% *w*/*v*, respectively), able to elicit an advantageous biological effect, was chosen to perform the following experiments.

### 3.2. Cell Cycle Analyses

**Figure 2:**
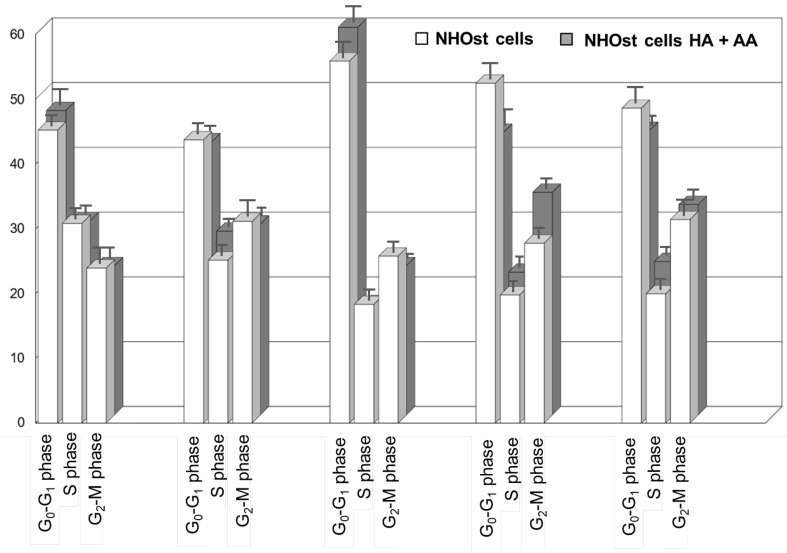
Cell cycle analysis resulting from flow cytometry experiments relevant for untreated NHOst cells and for NHOst cells exposed to HA+AA.

Figure 2 shows the cell cycle analyses of untreated NHOst cells, as well as of NHOst cells cultured in the presence of HA+AA solution. The exposure to the solution results in a faster cell cycle progression (also see Figure S1). Indeed, after 48 h in the presence of HA+AA solution, NHOst cells in the G_0_–G_1_ phase are significantly higher than in the untreated group (from 44% to 56% for untreated NHOst cells, from 42% to 60% in the presence of HA+AA, *p* < 0.05). Similarly, after 56 h, the shift from the S to G_2_-M phase is significantly higher (from 20% to 28% for the untreated NHOst cells; from 22% to 34% in the presence of HA+AA, *p* < 0.05), while the percentage of cells in the G_0_-G_1_ phase is significantly lowered (from 56% to 52% for the untreated NHOst cells; from 60% to 44% in the presence of HA+AA, *p* < 0.05). According to the literature, these findings suggest that Aminogam^®^ promotes the proliferation of NHOst cells, likely because of containing high-molecular-weight sodium hyaluronan molecules [17,28,29,30]. Furthermore, the enhancement of osteoblasts’ proliferation herein reported agrees with the results obtained in vivo, showing a faster consolidation of fractures and tissue healing in the presence of HA+AA [18,19].

### 3.3. Gene Expression Studies

**Figure 3:**
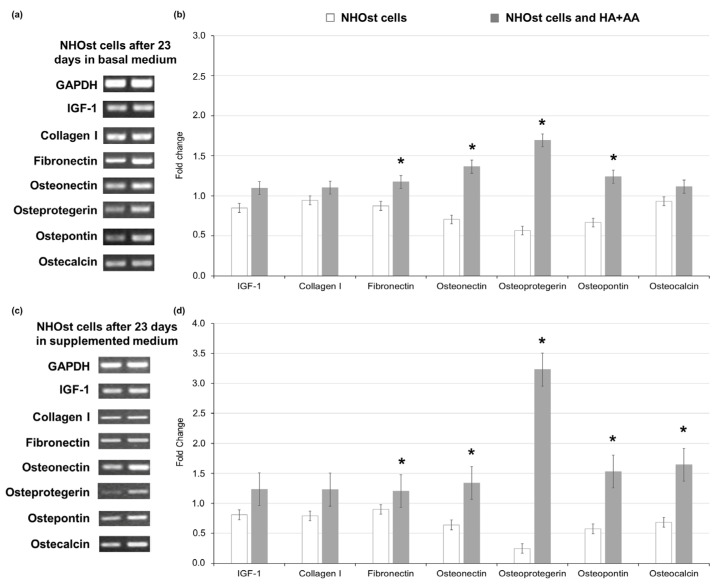
Gene expression of markers of osteoblasts’ differentiation, evaluated via RT-PCR after 23 days of culture in basal medium and in medium supplemented with 200 nM hydrocortisone-21-hemisuccinate and 7.5 mM β-glycerophosphate, with or without HA+AA solution. Panels (**a**) and (**c**) report the gel bands for the markers, assessed in NHOst cells cultured in basal (left) or supplemented medium (right). GAPDH represents the housekeeping gene. Panels (**b**) and (**d**) report the fold change values for each marker of osteoblasts’ differentiation (* *p* < 0.05).

The assessment of gene expression markers in NHOst cells exposed to HA+AA was performed via RT-PCR after 23 days of culture, comparing the obtained results with those related to untreated NHOst cells cultured in basal or supplemented medium (letter C vs. A in Figure 3). Considering GAPDH as the housekeeping gene, in NHOsts cells exposed to HA+AA in basal medium, an enhanced expression of adhesion proteins (i.e., fibronectin, osteopontin) and osteonectin, as well as of the main osteoclast-regulating protein, osteoprotegerin, is observed (Figure 3a,b). As far as the cells cultured in supplemented medium are concerned, the levels of osteoprotegerin, as well as those of genes associated with mineralization processes (i.e., osteocalcin, osteonectin and bone sialoproteins, such as osteopontin), are significantly higher in NHOst cells exposed to HA+AA (Figure 3c,d). The obtained results highlight that the combination of sodium hyaluronate and amino acids significantly improves NHOst cells’ differentiation in vitro, leading to mature osteoblasts expressing the late markers of the osteogenic process [31,32]. The proper cell maturation in the presence of HA+AA guarantees the deposition of the extracellular matrix, which is essential during tissue repair. In addition, the inhibition of osteoclasts’ activity through the pathway involving osteoprotegerin is responsible for the reduction in bone resorption, with subsequent recovery of bone mass observed in vivo [18,19,33].

### 3.4. Assessment of ALP Activity and Cell Morphology

**Figure 4:**
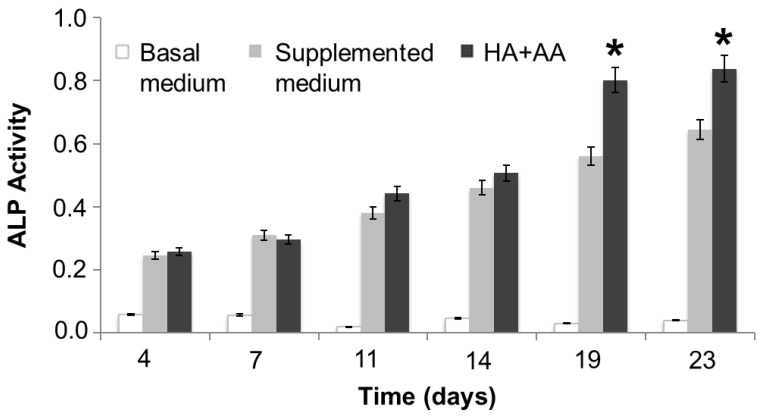
ALP activity assay. The reported data represent the means of three independent experiments. The HA+AA group is significantly different from the supplemented medium group after 19 and 23 days of culture (* *p* < 0.05).

**Figure 5:**
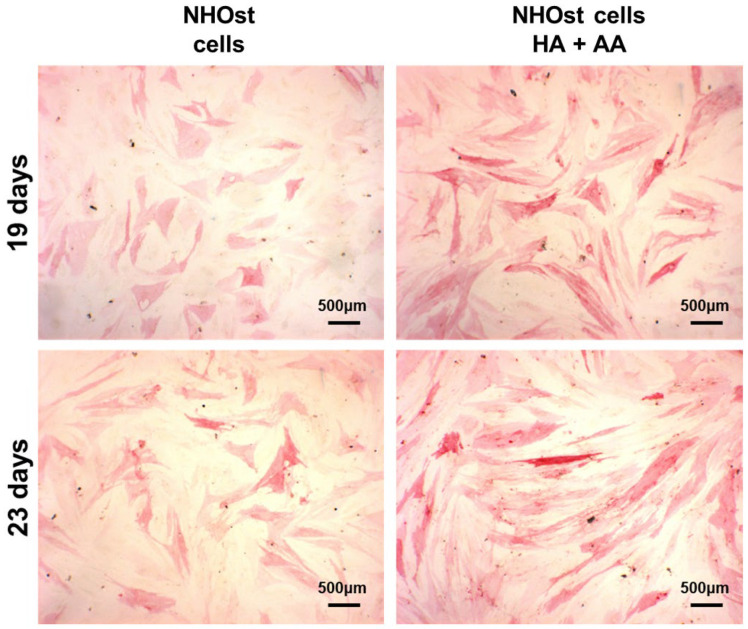
Representative images of ALP staining, performed after 19 or 23 days of culture, on untreated NHOst cells and on cells exposed to HA+AA.

The ALP activity has been evaluated in NHOst cells cultured in basal or supplemented medium, exposed or not to HA+AA solution, for up to 23 days (Figure 4). After 14 days of culture and up to 23 days, the presence of HA+AA elicits a significant enhancement of ALP activity (* *p* < 0.05), in agreement with the literature data which report an ALP activity increase as being indicative of early-stage osteogenesis [34]. The images obtained through ALP staining provide a representation of the same trend observed with the ALP activity assay (Figure 5). Indeed, the intensity of the red color increases over time, as well as in the presence of the HA+AA solution. The ALP staining also allows one to appreciate differences in cell morphology. Indeed, after 19 and 23 days, NHOst cells exposed to HA+AA increase in cell number and result in being more spread out and elongated as compared to control cells (also see Figure S2). In particular, by Day 19, untreated NHOst cells still display the cuboidal shape typical of low-density cultures. Conversely, in the presence of HA+AA, cells assume a fibroblastic spindle-shaped morphology, contacting each other via elongated cytoplasmic processes.

## 4. Discussion

In this work, the in vitro effects of HA+AA solution on NHOst cells have been investigated, explaining the mechanisms underlying the positive impact of HA+AA already observed in vivo. Other authors have described the effects of hyaluronate on osteoblast cells, highlighting an enhancement in cell proliferation, ALP activity and osteocalcin gene expression [35]. Meanwhile, other researchers have focused on the impact of selected amino acids on bone formation [36]. Herein, for the first time, the combination of HA+AA has been evaluated. After 3 and 4 days of culture, the MTT assay highlights the enhanced metabolic activity of NHOst cells cultured in the presence of HA+AA. Thus, the most advantageous HA+AA solution has been further tested with NHOst cells in vitro to shed light on the molecular machineries determining HA+AA effectiveness. The reported findings point out that, after 56 h in the presence of HA+AA, cell cycle progression is sped up, in particular from the S to G_2_-M phase. Furthermore, HA+AA also improves the expression of genes relevant to osteoblasts’ differentiation, even in basal medium. In addition, the ALP activity of NHOst cells increases significantly after 23 days of culture with HA+AA. Igarashi and coworkers pointed out the pivotal role of ALP in osteoblasts’ maturation, reporting its expression from the early stages of differentiation, with a continuous increase over 21 days of culture, until mineralization [37]. Therefore, our data are in agreement with the literature findings [38], although it must be taken into account that they refer to in vitro assessments. Indeed, a substantial gap between in vivo conditions and the experimental setup cannot be excluded [39]. For these reasons, the work has been carried out on primary osteoblasts, which could resemble more closely the real clinical scenario, as stated by Pagani and coworkers [40]. Cell lines could actually be easier to use and could provide more reproducible results. However, it has been reported that they might display an expression of genes coding for bone proteins (i.e., osteopontin) that are significantly different from that of primary osteoblast cells [41].

Beyond the limitations of in vitro cell culture experiments, this work focuses on the cellular and molecular response of NHOst cells to HA+AA. This combination positively affects the expression of several markers of osteoblasts’ differentiation and could be responsible for subsequent changes at the protein level, as well as at the final stages of new bone matrix deposition. Nevertheless, several cell types are involved in these processes (i.e., osteoclasts, osteoblasts, osteocytes and endothelial cells) and they should be studied as a whole complex system, i.e., the basic multicellular unit [42]. For this reason, further work will shed light on these points, dissecting the cross-talk between osteoblasts and osteoclasts in vitro.

## 5. Conclusions

The main findings of this work shed light on the mechanisms underlying HA+AA effectiveness reported in vivo, explaining the advantages of this formulation to treat injuries of bone and of the surrounding soft tissues. Future studies will investigate the cross-talk between osteoblasts and osteoclasts co-cultured in the presence of HA+AA, as well as its impact on models of bone diseases.

Taken together, the results presented in this work have demonstrated a positive in vitro effect of HA+AA solution on NHOst cells’ metabolism, proliferation and differentiation. Furthermore, the composition of the HA+AA solution allows one to prepare a wide range of formulations (i.e., injectable gel, spray and mouthwash), which could be valuable tools to face surgical and rehabilitation challenges in all clinical situations requiring bone regeneration.

## Figures and Tables

**Table 1:** Primer sequences exploited for gene expression studies.

mRNA	Primers	No. of Cycles
GAPDH	For: 5′-TTGGTATCGTGGAAGGACTCA-3′ Rev: 5′-TGTCATCATATTTGGCAGGTTT-3′	30
IGF-1	For: 5′-TGCCAATGTGGTGCTATTGT-3′ Rev: 5′-GAAAGGTGGTGGTGGCTAGA-3′	44
Collagen I	For: 5′-CTGGCAAAGAAGGCGGCAAA-3′ Rev: 5′-CTCACCACGATCACCACTCT-3′	25
Fibronectin	For: 5′-GCCTGGTACAGAATATGTAGTG-3′ Rev: 5′-ATCCCAGTGATCAGTAGGCTGGTG-3′	25
Osteonectin	For: 5′-GTGCAGAGGAAACCGAAGAG-3′ Rev: 5′-TCATTGCTGCACACCTTCTC-3′	27
Osteoprotegerin	For: 5′-GGCAACACAGCTCACAAGAA-3′ Rev: 5′-CTGGGTTTGCATGCCTTTAT-3′	32
Osteopontin	For: 5′-ACAGCCAGGACTCCATTGAC-3′ Rev: 5′-ACACTATCACCTCGGCCATC-3′	30
Osteocalcin	For: 5′-GGCAGCGAGGTAGTGAAGAG-3′ Rev: 5′-CTGGAGAGGAGCAGAACTGG-3′	30

## Data Availability

All reported data are available. Raw data are available from the corresponding author upon reasonable request.

## Supplementary Materials

The following supporting information can be downloaded at https://www.mdpi.com/article/10.3390/biomedicines11030751/s1: Figure S1: Flow cytometry analyses performed on untreated NHOst cells (a) or on NHOst cells exposed to HA+AA (b). Cell cycle phases have been studied using ModFit LT™ software (Verity Software House, Topsham, ME, USA); Figure S2: Percentage area covered by NHOst cells stained with Fast Red. Data are expressed as mean ± standard deviation and are referred to as three different optical fields per sample type.

## References

[1] Morgan E.F., Unnikrisnan G.U., Hussein A.I. (2018). Bone mechanical properties in healthy and diseased states. Annu. Rev. Biomed. Eng..

[2] Fang H., Deng Z., Liu J., Chen S., Deng Z., Li W. (2022). The Mechanism of Bone Remodeling After Bone Aging. Clin. Interv. Aging.

[3] Cui J., Shibata Y., Zhu T., Zhou J., Zhang J. (2022). Osteocytes in bone aging: Advances, challenges, and future perspectives. Ageing Res. Rev..

[4] Wu Y., Zhang X., Zhao Q., Tan B., Chen X., Liao J. (2020). Role of hydrogels in bone tissue engineering: How properties shape regeneration. JBN.

[5] Nallusamy J., Das R.K. (2021). Hydrogels and their role in bone tissue engineering: An overview. J. Pharm. Bioallied. Sci..

[6] Fakhari A., Berkland C. (2013). Applications and emerging trends of hyaluronic acid in tissue engineering, as a dermal filler and in osteoarthritis treatment. Acta Biomater..

[7] Rampal S., Jaiman A., Tokgöz M.A., Arumugam G., Sivananthan S., Singh R.S.J., Zazali S.B., Mohaddes M. (2022). A review of the efficacy of intraarticular hip injection for patients with hip osteoarthritis: To inject or not to inject in hip osteoarthritis?. Jt. Dis. Relat. Surg..

[8] Kim H., Jeong H., Han S., Beack S., Hwang B.W., Shin M., Seung S.O., Hahn S.K. (2017). Hyaluronate and its derivatives for customized biomedical applications. Biomaterials.

[9] Thompson C., Fuhrman M.P. (2005). Nutrients and wound healing: Still searching for the magic bullet. Nutr. Clin. Pract..

[10] Beckman M.J., Shields K.J., Diegelmann R.F. (2002). Collagen Metabolism. EBBE.

[11] Capodiferro S., Tempesta A., Bucci S., Maiorano E., Favia G., Limongelli L. (2020). Aminogam^®^ Gel Allows Faster Wound Healing after Oral Surgery by Formation of Mature Connective Tissue with Low Vascular Density and Reducing Inflammatory Infiltration. A Retrospective Study on 580 Cases with Histological and Confocal Laser Investigation. Appl. Sci..

[12] Nataliya K., Riccardo S., Canciani E., Toma M., Pellegrini G., Carmagnola D., Henin D., Dellavia C.P.B. (2017). Histomorphometrical evaluation of the effects of Aminogam® gel in oral healing process of post-surgical soft tissue. Ital. J. Anat. Embryol..

[13] Franco S., Miccoli S., Limongelli L., Tempesta A., Favia G., Maiorano E., Favia G. (2014). New dimensional staging of bisphosphonate-related osteonecrosis of the jaw allowing a guided surgical treatment protocol: Long-term follow-up of 266 lesions in neoplastic and osteoporotic patients from the university of Bari. Int. J. Dent..

[14] Colella G., Vicidomini A., Soro V., Lanza A., Cirillo N. (2012). Molecular insights into the effects of sodium hyaluronate preparations in keratinocytes. Clinical and Experimental Dermatology. Exp. Dermatol..

[15] La Gatta A., D’Agostino A., Schiraldi C., Colella G., Cirillo N. (2019). A biophysically-defined hyaluronic acid-based compound accelerates migration and stimulates the production of keratinocyte-derived neuromodulators. Cell. Adh. Migr..

[16] Favia G., Mariggiò M.A., Maiorano F., Cassano A., Capodiferro S., Ribatti D. (2008). Accelerated wound healing of oral soft tissues and angiogenic effect induced by a pool of aminoacids combined to sodium hyaluronate (AMINOGAM). J. Biol. Regul. Homeost. Agents..

[17] Mariggiò M.A., Cassano A., Vinella A., Vincenti A., Fumarulo R., Muzio L.L., Maiorano E., Ribatti D., Favia G. (2009). Enhancement of fibroblast proliferation, collagen biosynthesis and production of growth factors as a result of combining sodium hyaluronate and aminoacids. Int. J. Immunopathol. Pharmacol..

[18] Dipaola M., Digioia G., Armenio A., Pascone M. (2013). Use of AMINOGAM gel in hand fractures. G. Chir. JISA.

[19] Cosola S., Oldoini G., Boccuzzi M., Giammarinaro E., Genovesi A., Covani U., Marconcini S. (2022). Amino Acid-Enriched Formula for the Post-Operative Care of Extraction Sockets Evaluated by 3-D Intraoral Scanning. Int. J. Environ. Res. Public Health.

[20] Rajzer I., Menaszek E., Kwiatkowski R., Planell J.A., Castano O. (2014). Electrospun gelatin/poly (ε-caprolactone) fibrous scaffold modified with calcium phosphate for bone tissue engineering. Mat. Sci. Eng. C.

[21] De Giglio E., Bonifacio M.A., Cometa S., Vona D., Mattioli-Belmonte M., Dicarlo M., Ceci E., Fino V., Cicco S.R., Farinola G.M. (2015). Exploiting a new glycerol-based copolymer as a route to wound healing: Synthesis, characterization and biocompatibility assessment. Colloids. Surf. B.

[22] Ormerod M.G., Tribukait B., Giaretti W. (1998). Consensus report of the task force on standardisation of DNA flow cytometry in clinical pathology. Anal. Cell Pathol..

[23] Ji M., Zhao P., Cui Y., Li X. (2021). The Effect of Dexmedetomidine on Breast Cancer Cell Growth and Metastasis by Regulating the Expression of circRNA and Its Effect Mechanism. Acta Medica Mediterr..

[24] Correia C.R., Pirraco R.P., Cerqueira M.T., Marques A.P., Reis R.L., Mano J.F. (2016). Semipermeable capsules wrapping a multifunctional and self-regulated co-culture microenvironment for osteogenic differentiation. Sci. Rep..

[25] Schindelin J., Arganda-Carreras I., Frise E., Kaynig V., Longair M., Pietzsch T., Preibisch S., Rueden C., Saalfeld S., Schmid B. (2012). Fiji: An open-source platform for biological-image analysis. Nat. Methods.

[26] Sardella E., Mola M.G., Gristina R., Piccione M., Veronico V., De Bellis M., Cibelli A., Buttiglione M., Armenise V., Favia P. (2020). A synergistic effect of reactive oxygen and reactive nitrogen species in plasma activated liquid media triggers astrocyte wound healing. Int. J. Mol. Sci..

[27] R Core Team (2018). R: A Language and Environment for Statistical Computing. https://www.R-project.org/.

[28] Dovedytis M., Liu Z.J., Bartlett S. (2020). Hyaluronic acid and its biomedical applications: A review. Eng. Regen..

[29] Assmann V., Fieber C., Herrlich P., Hofmann M., Termeer C.C., Ahrens T., Simon J.C. (2001). CD44 is the Principal Mediator of Hyaluronic-Acid-Induced Melanoma Cell Proliferation. J. Investig. Dermatol..

[30] Wilkesmann S., Westhauser F., Fellenberg J. (2020). Combined fluorescence-based in vitro assay for the simultaneous detection of cell viability and alkaline phosphatase activity during osteogenic differentiation of osteoblast precursor cells. Methods Protoc..

[31] Safadi F.F., Barbe M.F., Abdelmagid S.M., Rico M.C., Aswad R.A., Litvin J., Popoff S.N. (2009). Bone structure, development and bone biology. Bone Pathology.

[32] Sasaki T., Watanabe C. (1995). Stimulation of osteoinduction in bone wound healing by high-molecular hyaluronic acid. Bone.

[33] Tong X., Gu J., Song R., Wang D., Sun Z., Sui C., Zhang C., Liu X., Bian J., Liu Z. (2018). Osteoprotegerin inhibit osteoclast differentiation and bone resorption by enhancing autophagy via AMPK/mTOR/p70S6K signaling pathway in vitro. J. Cell Biochem..

[34] Sengupta S., Park S.H., Patel A., Carn J., Lee K., Kaplan D.L. (2010). Hypoxia and Amino Acid Supplementation Synergistically Promote the Osteogenesis of Human Mesenchymal Stem Cells on Silk Protein Scaffolds. Tissue Eng. Part A.

[35] Huang L., Cheng Y.Y., Koo P.L., Lee K.M., Qin L., Cheng J.C.Y., Kumta S.M. (2003). The effect of hyaluronan on osteoblast proliferation and differentiation in rat calvarial-derived cell cultures. J. Biomed. Mater. Res. A.

[36] MacDonell R., Hamrick M.W., Isales C.M. (2016). Protein/amino-acid modulation of bone cell function. BoneKEy Rep..

[37] Igarashi M., Kamiya N., Hasegawa M., Kasuya T., Takahashi T., Takagi M. (2004). Inductive effects of dexamethasone on the gene expression of Cbfa1, Osterix and bone matrix proteins during differentiation of cultured primary rat osteoblasts. J. Mol. Histol..

[38] Wrobel E., Leszczynska J., Brzoska E. (2016). The characteristics of human bone-derived cells (HBDCS) during osteogenesis in vitro. Cell Mol. Biol. Lett..

[39] Rahmati M., Silva E.A., Reseland J.E., Heyward C., Haugen H.J. (2020). Biological responses to physicochemical properties of biomaterial surface. Chem. Soc. Rev..

[40] Pagani S., Liverani E., Giavaresi G., De Luca A., Belvedere C., Fortunato A., Leardini A., Fini M., Tomesani L., Caravaggi P. (2021). Mechanical and in vitro biological properties of uniform and graded Cobalt-chrome lattice structures in orthopedic implants. J. Biomed. Mater. Res. B Appl. Biomater..

[41] Ansari S., Ito K., Hofmann S. (2021). Cell Sources for Human in vitro Bone Models. Curr. Osteoporos. Rep..

[42] Kular J., Tickner J., Chim S.M., Xu J. (2012). An overview of the regulation of bone remodelling at the cellular level. Clin. Biochem..

