# Supplementary material for: In Vitro Evaluation of the Effects of Hyaluronic Acid and an Aminoacidic Pool on Human Osteoblasts
Source: Biomedicines. 2023 Mar 1;11(3):751. doi: 10.3390/biomedicines11030751 (PMC10045574; doi:10.3390/biomedicines11030751)
Supplement: Supplementary file 1 [file biomedicines-11-00751-s001.zip › biomedicines-2204804-supplementary.pdf]

biomedicines

Supplementary Materials

In vitro evaluation of the effects of hyaluronic acid and an aminoacidic pool on human osteoblasts

Maria Addolorata Bonifacio [1,†], Amalia Cassano [1,†], Alessandra Vincenti [1], Angela Vinella [1], Fabio Dell'Olio [2], Gianfranco Favia [2], and Maria Addolorata Mariggiò [1,*]

[1] Department of Precision and Regenerative Medicine and Ionian Area, University of Bari Aldo Moro Medical School, 70124 Bari, Italy; m.bonifacio@studenti.uniba.it; lianacassano@libero.it; a.vincenti2005@gmail.com; angela.vinella@uniba.it; mariaaddolorata.mariggio@uniba.it
[2] Department of Interdisciplinary Medicine, University of Bari Aldo Moro, Italy; gianfranco.favia@uniba.it; f.dellolio.odo@outlook.it
* Correspondence: mariaaddolorata.mariggio@uniba.it
† These authors equally contributed to the work.

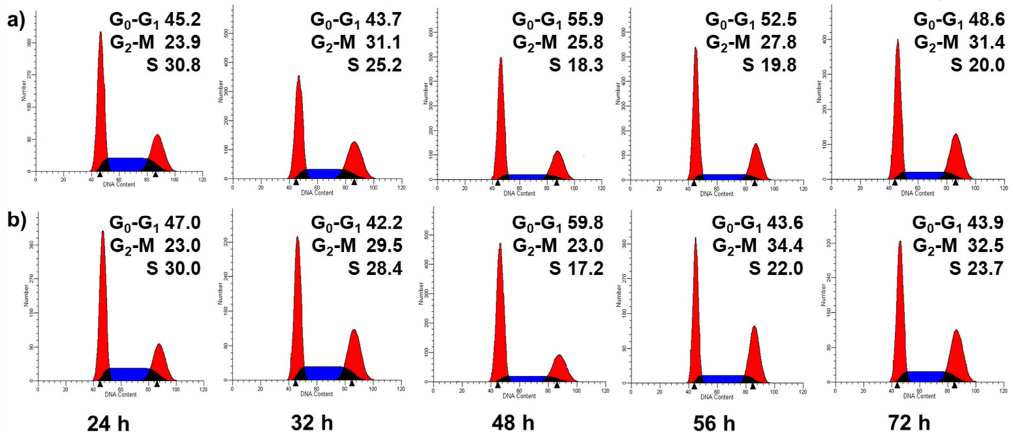

Figure S1. Flow-cytometry analyses performed on untreated NHOst cells (a) or on NHOst cells exposed to HA+AA. Cell cycle phases have been studied by ModFit LT™ software (Verity Software House, Topsham, ME, USA).

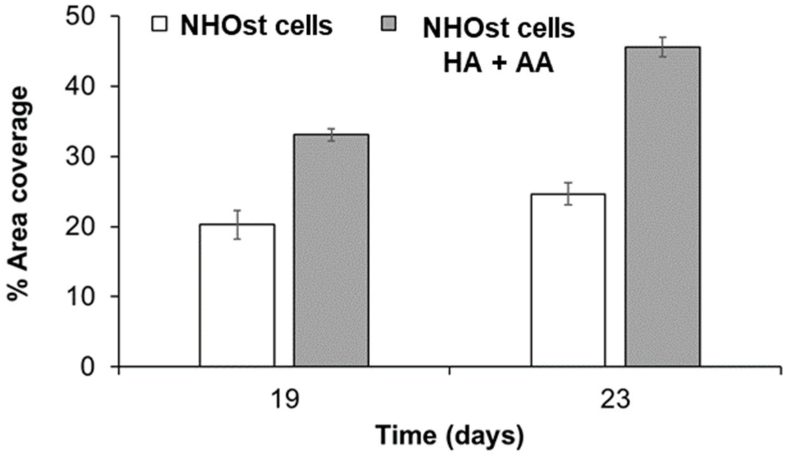

Figure S2. Percentage area covered by NHOst cells stained with Fast Red. Data are expressed as mean ± standard deviation and are referred to three different optical fields per sample type.

Citation: To be added by editorial staff during production.

Academic Editor: Firstname Lastname

Received: date
Revised: date
Accepted: date
Published: date

Copyright: © 2023 by the authors. Submitted for possible open access publication under the terms and conditions of the Creative Commons Attribution (CC BY) license (https://creativecommons.org/licenses/by/4.0/).
